# α-mannosidosis diagnosis in Brazilian patients with MPS-like symptoms

**DOI:** 10.1186/s13023-024-03419-z

**Published:** 2024-11-26

**Authors:** Maryana Marins, Marco Antonio Curiati, Caio Perez Gomes, Renan Paulo Martin, Priscila Nicolicht-Amorim, Joyce Umbelino da Silva Yamamoto, Vânia D’Almeida, Ana Maria Martins, João Bosco Pesquero

**Affiliations:** 1 Center for Research and Diagnosis of Genetic Diseases - Department of Biophysics, Universidade Federal de São Paulo, São Paulo, Brazil; 2 Inborn Errors of Metabolism Reference Center, Department of Pediatrics, Escola Paulista de Medicina, Universidade Federal de São Paulo, São Paulo, Brazil; 3 McKusick-Nathans Department of Genetic Medicine, Johns Hopkins University School of Medicine, Baltimore, MD USA; 4 Laboratory of Inborn Errors of Metabolism, Department of Psychobiology, Escola Paulista de Medicina, Universidade Federal de São Paulo, São Paulo, Brazil

**Keywords:** α-mannosidosis, Mucopolysaccharidosis, Lysosomal storage disease, MAN2B1, Hurler-like, MPS-like

## Abstract

**Background:**

α-mannosidosis is an inborn error of metabolism caused by the deficiency of the lysosomal enzyme α-mannosidase, which is encoded by the *MAN2B1* gene and inherited in an autosomal recessive manner. The impairment of affected individuals is multisystemic and very similar to the observed in some mucopolysaccharidosis (MPS) patients. The aim of this study was to search for α-mannosidosis cases in individuals with clinical suspicion of MPS without a confirmed diagnosis. Biochemical and molecular analysis were standardized by our group for this study. Two hundred and fifty samples from patients with clinical suspicion of MPS, but with inconclusive MPS biochemical and/or molecular analysis, were screened for α-mannosidase activity. Subsequently the *MAN2B1* gene was sequenced in samples from 53 patients by the Sanger method.

**Results:**

The measurement of enzymatic activity detected fifty-three samples with abnormal results, suggesting α-mannosidosis. Molecular analysis confirmed three affected families, which presented the nonsense variant p.Ser899Ter. This variant generates a premature stop codon in exon 22, resulting in a truncated protein with no residual enzymatic activity.

**Conclusion:**

In conclusion, this work brings data for the beginning of a genetic characterization of α-mannosidosis in the Brazilian population. It also shows that α-mannosidosis cases may be underdiagnosed due to the clinical similarity to MPS and the lack of information about this ultra-rare disease. Based on our data, we strongly recommend to all screening centers to consider α-mannosidosis testing together with screening for MPS as a tool for diagnosis to MPS-like phenotype individuals, since the phenotype similarity between these diseases poses a significant challenge for clinicians worldwide and often leads to the failure of the correct clinical diagnosis and treatment.

**Supplementary Information:**

The online version contains supplementary material available at 10.1186/s13023-024-03419-z.

## Background

α-mannosidosis (OMIM #248500) is a ultra-rare inborn error of metabolism, caused by the deficiency of lysosomal α-mannosidase enzyme [1], which was described first as Hurler-like syndrome [2]. Hurler is a type of mucopolysaccharidosis [3]. The first known human case was a boy with a Hurler-like phenotype described by the physician Öckerman in Lund, Sweden in 1967 [2]. After the boy died at 4yo, the results of his tissue biopsies showed large amounts of mannose. For this reason, the term “Mannosidosis” was suggested as the name of this disease and the case turned out to be α-mannosidosis [4]. Later in 1977, another atypical form of mucopolysaccharidosis was described, and turned out to be a α-mannosidosis case as well [5].

α-mannosidosis has not been described only in humans, but also in cattle, domestic cats, mice and guinea pigs [6–11]. In humans, α-mannosidosis is a multisystemic, progressive disease, and results mainly in facial and skeletal abnormalities, hearing impairment, intellectual disability, and immune deficiency [12, 13]. α-mannosidosis has been classically divided into three sub-types. Type 1 is a moderate form that presents slow progression, myopathy, and absence of skeletal abnormalities, clinically recognized after age 10. Type 2 is also a moderate form, presents slow progression, myopathy, but with the presence of skeletal abnormalities, clinically recognized before age 10. Type 3 is a severe form, presents central nervous system (CNS) involvement and quick progression with early death [12].

The prevalence of this disease varies from 1:500,000 to 1:1,000,000 [12–16]. The highest incidence found is 0,72:100.000 in Cuban population [17].

α-mannosidosis is an autosomal recessive disease, caused by pathogenic variants in *MAN2B1* (LAMAN) gene, which encodes the lysosomal enzyme α-mannosidase, responsible for the degradation of mannose rich oligosaccharides, and its absence leads to the accumulation of these substances in all tissues causing functional disturbances of cells [1, 12]. The *MAN2B1* gene is located on chromosome 19 (19p13.2-p13.11) and is composed by 24 exons spanning 21.5 kb [18–23]. So far, there are 185 pathogenic/probably pathogenic variants reported in HGMD data bank, consulted in September 2024 [24], being 102 missense/nonsense, 29 splicing substitutions, 27 small deletions, 20 small insertions/duplications and 7 gross deletions. In the α-mannosidosis variants database [25], there are 150 pathogenic variants described, including missense, nonsense, small and large deletions, small insertions and splice site variants. According to Varsome that compiles data from UniProt, ClinVar, VarSome Community and PubMed, there are in total 1427 classified variants in MAN2B1 gene, being: 339 pathogenic/likely pathogenic, 646 benign/likely benign and 442 of uncertain significance. Germany, United States and United Kingdom are countries in which more cases have been registered. Up to now, there are no cases reported in the Brazilian population [25].

The diagnosis of α-mannosidosis is based on the measurement of acid α-mannosidase activity, affected individuals usually present 5–15% of the normal activity observed in peripheral blood leukocytes. In addition to the biochemical test, molecular analysis of *MAN2B1* gene is also required to confirm the diagnosis. In this case, to be affected, the individual must present pathogenic variants in both alleles [12, 20]. As a screening tool, the urinary test to measure amounts of mannose-rich oligosaccharides is useful. Elevated urinary excretion of mannose-rich oligosaccharides can be suggestive of α-mannosidosis, but not a confirmation of the diagnosis [20].

In addition to monitoring the symptoms, the treatment of α-mannosidosis can be done with hematopoietic stem cell transplantation [1], which is indicated to preserve neurocognitive function and prevention of early death [21]. In 2018, the European Medicines Agency has authorized in the European Union the use of Lamzede (Velmanase alfa) for long-term enzyme replacement treatment (ERT) in adults, adolescents, and children with moderate form of α-mannosidosis [21]. Considering this possibility of treatment, it is paramount to develop a guideline for early recognition of α-mannosidosis, as previously stated by Guffon et al. [26], aiming to detect the most common signs and symptoms of the disease, according to the age of the patient. The success of the ERT means life quality for the patients and reaffirms the importance of more tools to avoid misdiagnosis due to the lack of information about α-mannosidosis, especially due to the MPS clinical features similarity. As our laboratory received suspected cases of MPS, some of them without the confirmed diagnosis, we have investigated the possibility of α-mannosidosis in this group.

## Methods

### Ethical aspects

The medical records and samples for retrospective studies was approved by the Ethics Committee in Research of Universidade Federal de São Paulo (CEP: 0791–2016).

## Biochemical assay

### Control group and sample collection

Fifty healthy individuals, aged between 20 and 55 yo (age mean 31yo) volunteered for a control group, for the determination of normal activity of the α-mannosidase enzyme. Fresh whole blood samples were collected in heparin tubes, a blood drop was transferred to a paper filter card Whatman™ 903^®^ (dried blood spot - DBS), filling the marked circles and dried at room temperature for at least 4 h. After drying, the DBS was stored at 4ºC until the measurement of enzyme activity.

## Study group and sample collection

Two hundred and fifty patients samples previously received in our laboratory for MPS screening without confirmed diagnosis, were selected for analysis of α-mannosidase activity assay in DBS, described below. All blood samples were previously collected in heparin tubes and stored on a paper filter card Whatman™ 903^®^ (dried blood spot – DBS), filling the marked circles and dried at room temperature for at least 4 h. After drying, the DBS was stored at 4ºC until the measurement of enzyme activity.

### Standardization of α-mannosidase activity assay

The asssay was performed to detect in vitro α-mannosidase activity, using the fluorescent substrate 4-MU-αD-mannopyranoside (Sigma^®^) by measuring the released 4MU fluorochrome as previously described [27, 28] and adapted by our group as follows. The reaction was done in 96 wells microplate (Nunclon Delta White Microwell SI – Nunc Brand Products) and fluorescence was measured with a spectral fluorimeter (Spectra Max M2 – Molecular Device, USA). Assays were performed with three 1.5-mm punches, 0.9 µL of blood/each, in duplicate for each DBS and 1.5-mm punches without sample from the same cards served as blanks, being one punch per well, two for the DBS and one for the blank. Thirty µL of MilliQ water was added to the blank wells. To the DBS sample wells, 30 µL of MilliQ water and 30 µL of the substrate 1.5 mM diluted in citrate/phosphate buffer 0.2 M pH 3,8 were added. Microplate was sealed and incubated for 2 h at 37 °C, shaking at 45 rpm. Substrate solution was also incubated separately in the same reaction. After incubation, the microplate was centrifuged at 265 g and the reaction was stopped with Glycine-Sodium Carbonate buffer 0.17 M pH 12.0 in all wells. Thirty µL of the substrate solution were added to the blank wells and they were separately incubated. Fluorescence readings were performed at 365 nm (excitation) and 450 nm (emission). A standard curve of 4-methylumbelliferone (4-MU) was prepared and the enzymatic activity was calculated based on the straight line equation and enzyme activity calculation through the equation y = a + bx, being “y” the fluorescence (mean of readings – blank), “x” sample concentration, “a” and “b” linear and angular coefficients, respectively on the blood volume on the incubation time: Y = a + bx /(0.9 µl/1000)/2 h. Fluorescence readings were corrected for blanks and results were compared with those of a 4MU calibrator prepared in stop buffer. Enzyme activity was expressed as nmol substrate hydrolyzed per mL of blood per hour (nmol/mL/hour).

The measurement of β-galactosidase enzymatic activity was performed as a positive control for the quality of the DBS. This procedure is part of our routine protocol for controlling all enzymatic activity assays in our screening center established by Muller et al. (2010) [28]. For β-galactosidase activity, cutoff values > 6 nmol/mL/h are considered normal and, therefore, the sample is considered adequate [28].

### Statistical analysis

Komolgorov-Smirnov test was used to evaluate the enzyme activity distribution in the control samples. Student’s t-test was use to evaluate the mean obtained between both genders within healthy volunteers.

## Molecular analysis

### Subjects

From the two hundred and fifty patients screened for *α*-mannosidase activity in the DBS, 53 samples were selected and submitted to the *MAN2B1* gene sequencing. Of the 53 patients, 43 were male and 10 were female, aged between < 1yo to 65 yo (age mean 10yo), their description and clinical manifestations are presented in the suplemmentary material (Table S1).

### Genomic DNA extraction

Genomic DNA extraction was performed with QiaAmp mini blood kit (Qiagen™) on the QIAcube (Qiagen™), following the manufacturer’s instructions.

### MAN2B1 gene sequencing

The twenty-four exons of *MAN2B1* gene were amplified with GoTaq Master Mix Kit (Promega). Primers (Exxtend) design and PCR conditions were standardized by our group and are described in the supplementary material (Tables S2 and S3). Sequencing was performed with BigDye Terminator v3.1 Cycle Sequencing Kit (Life Technologies, CA, USA) following the manufacturer’s instructions, in the ABI Prism 3500xl Genetic Analyzer sequencer (Life Technologies). The twenty-four exons and their proximal intronic regions were sequenced with 100% of coverage of the coding region of MAN2B1 gene.

### Data analysis

Sequences were analyzed with software Geneious (10.2.6 version) using the reference sequence NG_008318 (http://www.ncbi.nih.gov). The variants found were analyzed in silico and consulted on Varsome database (http://www.varsome.com), dbSNP database (single-nucleotide polymorphism, ncbi.nlm.nih.gov/projects/SNP/), Clinvar (https://www.ncbi.nlm.nih.gov/clinvar/), *α*-mannosidosis Mutation database (https://apex.jupiter.no) and HGMD (Human Genome Mutation Database professional, biobaseinternational.com/product/hgmd). Population frequency was consulted on gnomAD (https://gnomad.broadinstitute.org/). To predict the impact of the varints on the structure and function of the resulting protein we used the prediction softwares: CADD (https://cadd.gs.washington.edu/), Polyphen2 (genetics.bwh.harvard.edu/pph2) and Mutation Taster (mutationtaster.org). Splicing impact of intronic variants were analyzed on the web-software Human Splicing finder. ACMG standards and guidelines (American College of Medical Genetics and Genomics and the Association for Molecular Pathology) were used to classify variants according to the interpretation of sequence variants.

### Human lysosomal α-mannosidase modelling

Human lysosomal *α*-mannosidase model was generated by homology to the crystal structure of golgi *α*-mannosidosis II (PDB:3BUB) [29]. Nest routine from Jackal package was used to build the model (Jason Z. Xiang, Columbia University, New York, NY, USA). Primary sequence from Human lysosomal *α*-mannosidase and golgi *α*-mannosidosis II were aligned with software ClustalX 2.0 [30]. Struture analysis and ray-traced screenshots were performed with software Yasara (YASARA Biosciences, Viena, AUT).

## Results

### Biochemical α-mannosidase activity assay

For determination of normal activity of the α-mannosidase enzyme, 50 DBS samples from healthy and unrelated individuals were analyzed. Results of the measurement of enzyme activity are expressed as nmol substrate hydrolyzed per mL of blood per hour (nmol/mL/hour) and presented in Table 1. We considered only the results of the samples with < 20% coefficient variation between the duplicates (intra-test). The analysis of the enzyme activity in the control group showed a normal distribution of α-mannosidase activity in DBS of healthy individuals. Considering the statistical results, with the confidence interval and the minimum value obtained, the cutoff value > 7.24 nmol/mL/h was established for α-mannosidase activity in DBS of healthy individuals. β-galactosidase activity were adequate for all samples of this group.

**Table 1:** α-mannosidase activity in DBS of healthy individuals (*n* = 50, male and female, mean age 31yo)

Activity (nmol/mL/h)	
Mean	16.12
Standard deviation	8.15
Median	14.06
Confidence interval (95%)	13.86–18.38
Maximum	47.10
Minimum	7.24

### α-mannosidase activity assay for study group

250 DBS samples previously received for MPS screening without confirmed diagnosis were selected for the study group. Among the 250 evaluated samples analyzed, 129 samples presented normal results for α-mannosidase and β-galactosidase (positive assay control) activities and therefore the *MAN2B1* gene was not sequenced. The others 121 samples presented abnormal results for α-mannosidase activity, however only 53 samples showed normal values for β-galactosidase activity or had enough DNA for sequencing and therefore the *MAN2B1* gene was sequenced. The remaining samples were excluded from sequencing because the measurement of enzyme control activity tested was unfeasible [30] or there was an insufficient amount of DNA.

### Molecular analysis

From the 53 samples selected for molecular analysis, we found three different families affected by the same nonsense variant (p.Ser899Ter). We also detected 34 variants in the *MAN2B1* gene among the analyzed samples, 14 in the coding region and 21 in the non-coding region. The description and in silico analysis of the exonic and intronic variants are show in the Tables 2 and 3, respectively.

**Table 2:** Description of the exonic variants found in the *MAN2B1* gene in the 53 MPS-like patients analysed

Location	Nucleotide change	Amino acid change	Type of alteration	Nr. of patients	Nr. of alleles	Nr. of Homozygotes	dbSNP ID	Clinvar	ACMG classification	gnomAD Exomes
Exon 5	c.747 C > T	p.Thr249=	synonymous	3/53	3/106	0/53	rs61737536	Benign	(LB)	0.0133
Exon 6	c.832 C > G	p.Leu278Val	missense	23/53	29/106	6/53	rs1054486	Benign	(B)	0.251
Exon 7	c.935 C > T	p.Thr312Ile	missense	32/53	38/106	7/53	rs1054487	Benign	(B)	0.332
Exon 7	c.1010G > A	p.Arg337Gln	missense	29/53	31/106	2/53	rs1133330	Benign	(B)	0.307
Exon 10	c.1238 A > G	p.Asn413Ser	missense	10/53	11/106	1/53	rs35836657	Benign	(B)	0.0901
Exon 12	c.1441G > T	p.Ala481Ser	missense	4/53	4/106	0/53	rs34544747	Benign	(B)	0.0262
Exon 13	c.1540G > A	p.Val514Ile	missense	1/53	1/106	0/53	rs759767616	(VUS)	(VUS)	< 0.0001
Exon 16	c.2006 C > T	p.Pro669Leu	missense	2/53	2/106	0/53	rs75029862	Benign	(LB)	0.0092
Exon 18	c.2221G > A	p.Gly741Arg	missense	2/53	2/106	0/53	rs61234887	Benign	(B)	0.0043
Exon 18	c.2260G > A	p.Glu754Lys	missense	1/53	1/106	0/53	rs141212446	Conflicting	(LB)	0.0012
Exon 19	c.2310 C > T	p.Pro770=	missense	4/53	4/106	0/53	rs35880640	Likely benign	(B)	0.0126
Exon 21	c.2640T > C	p.Asn880=	synonymous	1/53	1/106	0/53	rs763786776	Likely benign	(LB)	0.0001
Exon 22	c.2696 C > A	p.Ser899Ter	nonsense	4/53	8/106	4/53	rs767323371	Pathogenic	(P)	0.0004
Exon 23	c.2865G > C	p.Thr955=	synonymous	1/53	1/106	0/53	rs148108322	Benign	(B)	0.0028

Genomic reference GRCh37, hg19 | NM_000528. Legends: (LB): Likely Benign. (B): Benign. (VUS): Uncertain Significance. (P): Pathogenic

**Table 3:** Description of the intronic and non-coding region variants found in the *MAN2B1* gene in the 53 MPS-like patients analysed

Location	Nucleotide change	Nr. of patients	Nr. of alleles	Nr. of Homozygotes	dbSNP ID	Clinvar classification	ACMG classification	gnomAD Genomes
Intron 6	c.910–73 A > G	30/53	38/106	8/53	rs2303731	Benign	Benign	> 0.005
Intron 7	c.1026 + 50 C > T	1/53	1/106	0/53	rs116003721	(-)	Benign	< 0.005
Intron 7	c.1026 + 108T > C	1/53	1/106	0/53	rs117586044	(-)	Likely benign	> 0.005
Intron 10	c.1309 + 34 C > A	22/53	23/106	1/53	rs34324185	Benign	Benign	< 0.005
Intron 10	c.1309 + 204 A > C	1/53	1/106	0/53	rs527270345	(-)	VUS	< 0.005
Intron 10	c.1310–22 C > T	34/53	42/106	10/53	rs73002392	Benign	Benign	> 0.005
Intron 11	c.1420-40G > C	3/53	3/106	0/53	rs112072150	Benign	Benign	> 0.005
Intron 13	c.1645–220 C > T	1/53	1/106	0/53	rs115743265	Likely benign	Benign	> 0.005
Intron 13	c.1645–336 A > C	3/53	4/106	1/53	rs111629860	(-)	Benign	> 0.005
Intron 13	c.1645–311 C > T	1/53	1/106	0/53	rs111352858	(-)	Benign	< 0.005
Intron 16	c.2046 + 184 A > G	18/53	30/106	12/53	rs17476839	Benign	Benign	> 0.005
Intron 18	c.2267 + 3G > C	1/53	1/106	0/53	rs28639634	Benign	Benign	< 0.005
Intron 18	c.2267 + 8dup	1/53	1/106	0/53	rs572289342	Conflicting	Benign	< 0.005
Intron 18	c.2267 + 63 A > G	38/53	53/106	15/53	rs3815914	Benign	Benign	< 0.005
Intron 18	c.2267 + 216 A > G	32/53	43/106	11/53	rs8102193	Benign	Benign	> 0.005
Intron 18	c.2268-45T > G	25/53	34/106	9/53	rs12984441	Benign	Benign	> 0.005
Intron 20	c.2437–23 C > A	1/53	1/106	0/53	rs189469608	(-)	Likely benign	< 0.005
Intron 23	c.2923 + 50G > A	1/53	1/106	0/53	rs59556856	Likely benign	Benign	> 0.005
Intron 23	c.2924-152G > A	5/53	5/106	0/53	rs61161691	Benign	Benign	> 0.005
3’UTR	c.*69C > T	3/53	3/106	0/53	rs972047517	(-)	Likely benign	< 0.005
3’UTR	c.*84A > T	1/53	1/106	0/53	(-)	(-)	VUS	< 0.005

Genomic reference GRCh37, hg19 | NM_000528. Legends: VUS: Uncertain Significance. (-) no entry

### Affected families

**Fig. 1:**
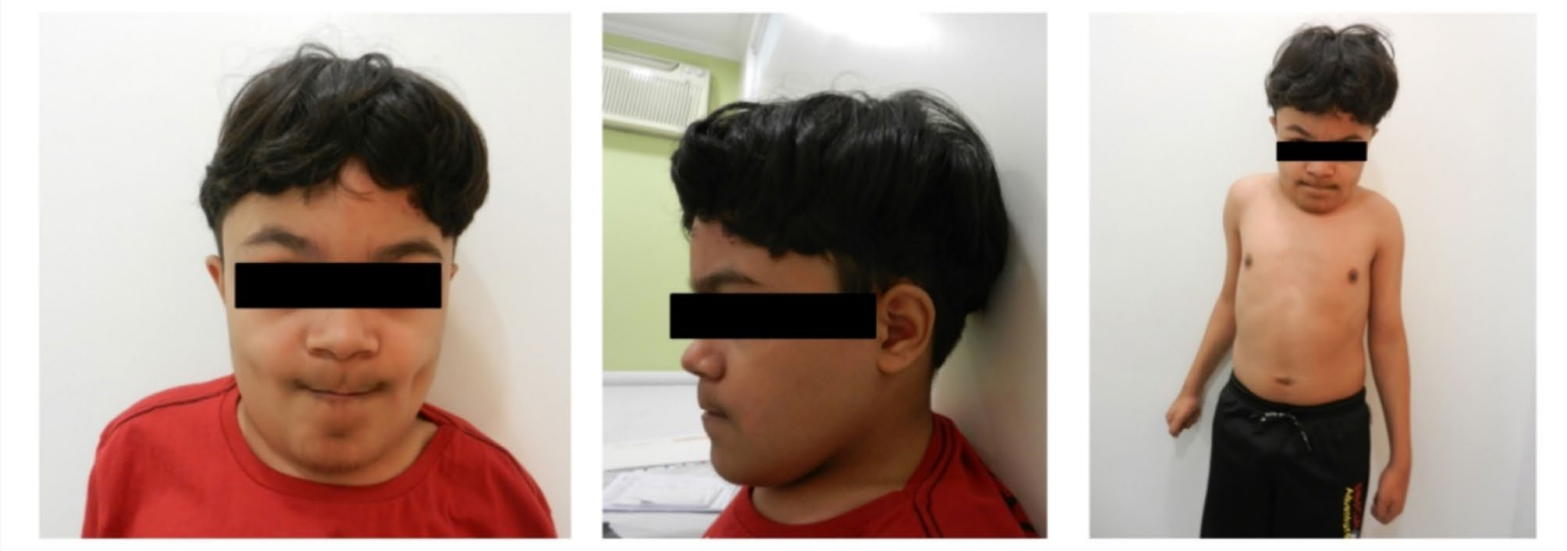
Affected patient (P1) from family A

We detected three affected families, these results are summarized in the Table 4. Family A originates from the state of Piaui (PI), Northeast region of Brazil. The affected patient (P1) is 22 yo, male and born from a consanguineous relationship, Fig. 1. P1 presented adequate motor development with significant speech delay (started to speak full phrases at 5 years old), significant learning disability in school and never was able to be alphabetized. Due to the developmental delay, he started follow-up in a neurology clinic, when syndromic intellectual disability was suspected. At the age of 15 he started to present neurodevelopmental regression with speech regression and gait regression. At 21 years old he presented ganglionar tuberculosis due to the immunodeficiency. At 22 years old the patient stopped speaking. Brain MRI presented cortical and cerebellar atrophy, and T2/FLAIR hyperintensities in the periventricular white matter, no-specific. Abdominal USG presented moderate hepatosplenomegaly. uGAGs and enzymatic activities for MPS I, II, and VII were normal. Oligossacharides and sialosaccharides chromatography showed high levels of mannose-rich saccharides in the urine and enzymatic activity of *α*-mannosidase in the blood was undetectable. Molecular analysis of *MAN2B1* gene presented the homozygous pathogenic variant p.Ser899Ter, confirming the diagnosis. Familiar segregation showed that both his parents carry the variant p.Ser899Ter in heterozygosity. Family B originates from the state of Paraíba (PB) also Northeast region of Brazil. The affected patient (P2) male, 6yo when MPS suspicious was first made by the clinician, and characteristic signs of the syndrome were described in his records. At that time, searching for an MPS diagnosis, the enzymatic activity assay was performed for MPS types I, II, IVA, VI including GAG test. All of the results did not confirm the diagnosis of MPS. Due to the technical limitations related to the long storage time we could not perform the enzymatic assay for α-mannosidosis. Molecular analysis detected the homozygous pathogenic variant p.Ser899Ter in the *MAN2B1* gene, confirming the diagnosis. We could not access more details of his clinical manifestations, since we worked with old records, considering this study was also retrospective.

**Table 4:** Summary information for affected patients

Family ID	Patient Code	Gender	Age at onset of symptoms	α-mannosidase activity	MAN2B1 variant	Zygosity	Family origin
A	1	Male	> 1yo	null	p.Ser899Ter	homozygosity	PI, Northeast of Brazil
B	2	Male	6yo*	N/A	p.Ser899Ter	homozygosity	PB, Northeast of Brazil
C	3	Male	2yo	null	p.Ser899Ter	homozygosity	SP, Southeast of Brazil
	4	Female	> 1yo	null	p.Ser899Ter	homozygosity	

*As we could not access his records, this age refers to the patient’s age when the sample was received by our center

Null: α-mannosidase activity was null, no residual activity of the enzyme was detected

N/A: not accessed due to the degradation of the sample

PI: Piauí State

PB: Paraíba State

SP: São Paulo State

Family C originates from the state of São Paulo (SP), Southeast of Brazil. There are two affected patients from this family, P3 and P4. P3 is 34 yo, male, presented adequate motor development with significant speech delay (started to speak full phrases at 4 years old), significant learning disability in school but was able to be alphabetized. Due to the developmental delay, he started follow-up in a neurology clinic, when MPS was suspected. At the age of 18 the patient started to present neurodevelopmental regression, forgot how to perform daily activities like answering the phone or purchasing groceries. At 22 years old he started with visual and auditory hallucinations and paranoid thoughts, and further regression was noticed after this episode. At 25 years old the patient stopped walking and soon after could not speak anymore. Currently, the patient is fully dependent for all daily living activities and started to present dysphagia to solid food. Brain MRI presented cortical and cerebellar atrophy, and T2/FLAIR hyperintensities in the white matter of the posterior horn of lateral ventricles, inespecific. Abdominal USG presented moderate hepatosplenomegaly. uGAGs and enzymatic activities for MPS I, II, and VII were normal.

P4 is 29 yo, female with adequate motor development and significant speech delay (first words at 4 years old). She presented significant learning disability (could not be alphabetized). At 27 years, patient started to present gait impairment (ataxic-hemiparetic), coordination impairment and heteroagressivity. At 28 years she presented further neurologic regression, could not shower or eat without assistance. Brain MRI presented cortical and cerebellar atrophy, and T2/FLAIR hyperintensities in the periventricular white matter, inespecific. Abdominal USG presented moderate hepatosplenomegaly. uGAGs and enzymatic activities for MPS I, II, and VII were normal. For both patients, oligossacharides and sialosaccharides chromatography showed high levels of mannose-rich saccharides and blood enzymatic activity of *α*-mannosidase was undetectable. Molecular analysis of MAN2B1 gene detected the homozygous pathogenic variant p.Ser899Ter, confirming the diagnosis.

Due to the impossibility of recruiting their parents, the family segregation analysis was not performed.

### Human lysosomal α-mannosidase modelling

**Fig. 2:**
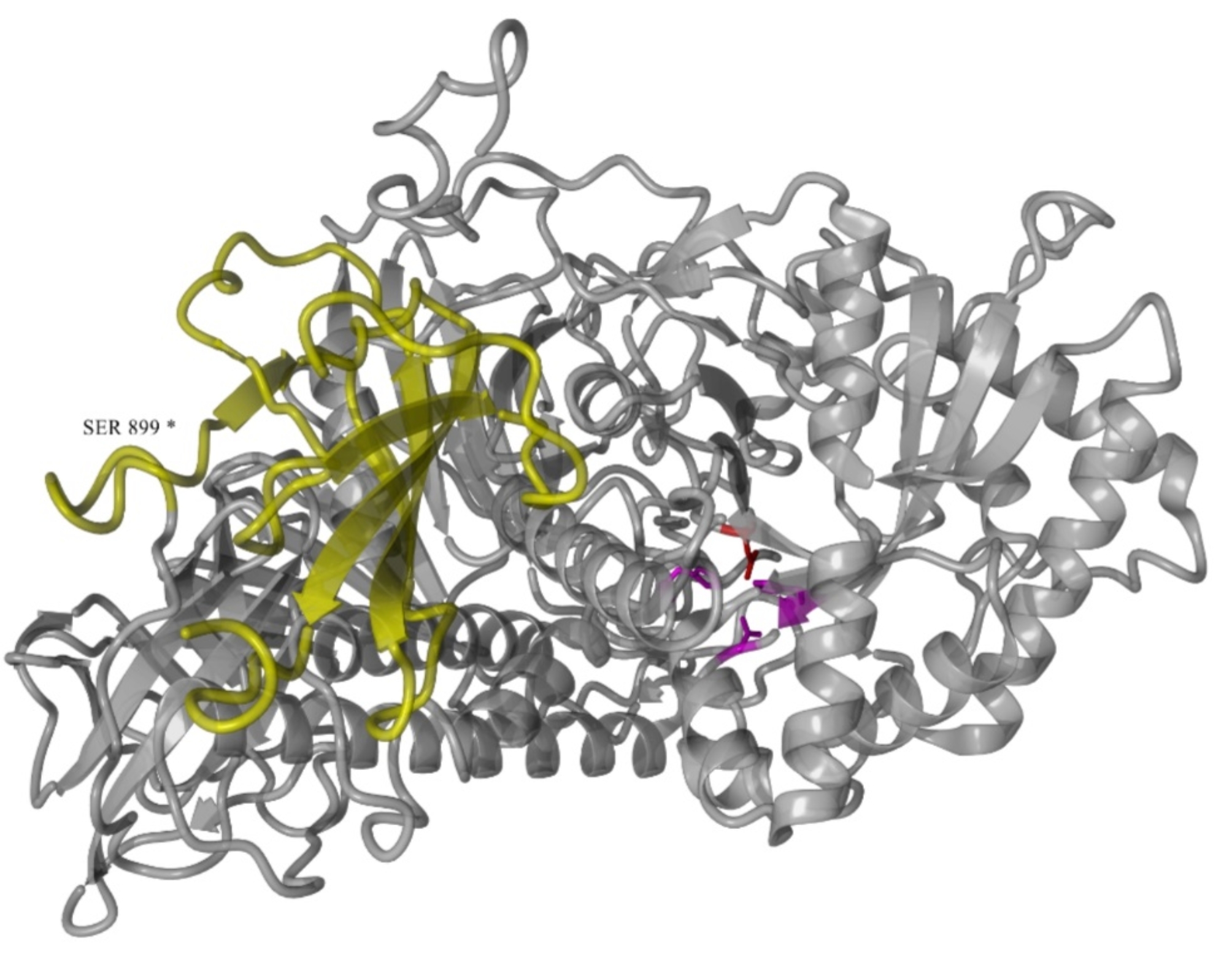
Human lysosomal **α**-mannosidase model

The picture of the human lysosomal α-mannosidase structure is shown in the Fig. 2. It represents the human lysosomal α-mannosidase modelling, yellow segment shows the deletion caused by the p.Ser899Ter mutation, which is the pathogenic variant found in four patients of this study. Purple residues represent zinc binding sites (His72, Asp74, and His446) and red represents active site residue Asp196.

## Discussion

This work is the first study to investigate the presence of α-mannosidosis in the Brazilian population. The finding of four affected patients, belonging to three families, out of 250 samples from patients with MPS suspicion supports our hypothesis that α-mannosidosis is an underdiagnosed disease in our country. We believe the reason for the underdiagnoses is the lack of information about this disease and clinical manifestations similarity between MPS and α-mannosidosis, which becomes a challenge for the clinicians worldwide to suspect α-mannosidosis.

As an example of the diagnostical challenge, we can cite the time that took to finally establish the diagnosis of these patients. All samples included in this study came to our center first to investigate MPS, the first α-mannosidosis diagnosis for the patient from family A took almost 10 years.

Regarding to the affected patient from family B, we could not access details of his clinical manifestations, since we worked with old records, considering this study was retrospective. Three years after the sample arrived at our laboratory, we were able to determine the correct diagnosis by using the molecular analysis. For the two affected patients from family C, we could establish the diagnosis faster, since they had been included in this study recently.

All affected patients of this study presented the same homozygously pathogenic variant p.Ser899Ter (c.2696 C > A). Until now, there are no published functional studies for this variant, however it is classified as pathogenic according to Clinvar and the ACMG criteria (PVS1, PM2, PP5), as it promotes a premature stop codon in the transcription of mRNA, resulting in a truncated protein causing the deficiency or inactivity of α-mannosidase enzyme. According to gnomAD browser the allele frequency of this variant is about 0.0004 for reference hg19 and 0.0008 for reference hg38, which is extremely low. There is no report of homozygous individuals for this variant. Also, both references shows that this variant was detected only in the European (non-Finnish) population. As we found no report in the literature of α**-**mannosidosis cases in the Brazilian population, it is still unclear whether this variant could be a founder in Brazil or could be brought to Brazil as result of the immigration of the Europeans to our country. Based on the migratory history of populations, the distribution of genetic variants can exhibit distinct frequencies depending on the ethnic and geographic context [31]. Brazil experienced significant European immigration, particularly in the 19th and 20th centuries, with groups coming from Portugal, Italy, Germany, among others. Moreover, studies on the genetics of Brazilian populations often reveal a complex interaction with European ancestries [32]. However, it is also possible that this variant originated in Brazil. It is known that the high rate of endogamy in some regions may favor the segregation of rare variants [33], this event still occurs in various regions of Brazil. As an example, family A in this study includes an affected patient who is born of a consanguineous marriage. Another point to consider is the geographic origin, with an incidence concentrated in the Northeast region of Brazil, as two of the three affected families are from that same region. Therefore, the origin of the p.Ser899Ter variant remains unclear, potentially resulting from either European migration or local processes of Brazilian genetic evolution.

Table 2 shows two exonic variants c.1540G > A and c.2260G > A, with conflicting classifications between Clinvar, ACMG criteria, Polyphen2 and Mutation Taster software’s prediction analysis. Both with low population frequency (< 0.005) according to gnomAD and therefore potentially relevant for future studies. The c.1540G > A variant is in a potentially conserved gene region according to the CADD score (2.308) and it is classified as uncertain significance in Clinvar as well as according to the ACMG criteria (PM2) with low population frequency (< 0.0001) and a lower allele frequency than that expected for the disease (0.00112) according to gnomAD. Functional tests are needed to evaluate this variant in homozygosity to conclude its pathogenicity. The c.2260G > A variant is classified as benign according to the ACMG criteria (BS1, BS2 and BP6), but there are conflicts of interpretation regarding its pathogenicity in Clinvar. Despite a low population frequency (< 0.005), the variant’s allele frequency is higher than that expected for the disease, and it was detected in homozygosity in some healthy individuals according to gnomAD. These findings suggest a benign classification for this variant.

Of the 21 variants described in Table 3, three could not be classified as probably benign or benign, considering only in silico analysis. Of these, the c.1309 + 204 A > C and c.*84A > T variants are classified as uncertain significance according to the ACMG criteria (PM2 and BP7), with low population frequency on gnomAD Genomes (< 0.005). Considering that both variants are non-coding and not located in a splice region, they may not be relevant as they have no splice-altering consequence. Still in Table 3, the c.2267 + 8dupG variant is classified as benign according to the ACMG criteria (BS2, BP4 and BP6), however there are conflicts of interpretation about its pathogenicity in Clinvar. In addition to conflicts in Clinvar for this variant, in silico analysis in the Human Splicing Finder prediction software indicates a potential impact on the splicing mechanism, in addition to a low frequency in agreement with the gnomAD Genomes (< 0.005). All the patients carried these variants in heterozygozity.

Due to the retrospective nature of the study, we had some limitations, such as the lack of patients’ data, resulting in a poor clinical detail of some patients. In addition, a small sample size and sample long storage time were limitations. Despite all these limitations, we have contributed to the standardization of the molecular and enzymatic assays for a rapid α-mannosidosis diagnosis to implement in our screening program. Using the enzymatic assay, we tested 250 blood samples from Brazilian patients with MPS-like phenotype, with negative MPS test results, received from 2015 to 2022. As a result, we detected four positive cases, being three positive cases of α-mannosidosis, confirmed by enzymatic assay and genetic testing and one that could be confirmed only by the genetic testing. In addition, there are 68 samples which could still be tested by molecular analysis or enzymatically in fresh DBS samples, to verify low results obtained and thus confirm or not the hypothesis of mannosidosis for these patients.

Our findings corroborate data from other groups, such as studies from Cuba, the Middle East and Europe, which also showed a higher frequency of α-mannosidosis than 1 in 500,000 live births, as shown in the literature [17, 22 and 23]. The highest α-mannosidosis incidence was confirmed in Cuba, being 0.72 in 100,000 live births. These numbers were confirmed by a study performed between 1990 and 2005, involving around 2 million samples with the aim of determining the occurrence of LSDs in the Cuban population [17].

Recently, a study tested 1010 MPS-like phenotype samples from Middle East and Europe, and of the 1010 samples tested, four were positive for α-mannosidosis [22]. Another study from Turkey population detected one positive case of α-mannosidosis out of 42 tested samples from suspicious MPS individuals [23].

## Conclusions

Taken together, these findings may suggest a significant higher frequency than 1:500,000 to 1:1,000,000 [12–16] for α-mannosidosis not just in Brazil but all over the world. In conclusion, our data represent a profile of variants in the *MAN2B1* for α-mannosidosis diagnosis in the Brazilian population, since this is the first initiative related to α-mannosidosis detection in our population, which can be one more tool to avoid misdiagnosis due the lack of information about this disease in our country. Therefore, based on our data, we strongly suggest to all screening centers to consider α-mannosidosis testing as a differential diagnosis to MPS-like phenotype, since the phenotype similarity between these diseases poses a significant challenge for clinicians worldwide and often leads to the failure of the correct clinical diagnosis and treatment.

## Electronic supplementary material

Below is the link to the electronic supplementary material.

Supplementary Material 1

## Data Availability

The datasets used and/or analyzed during the current study are available from the corresponding author on reasonable request and partially available at the link: URI. http://repositorio.unifesp.br/handle/11600/50529.

## Abbreviations

MPS: Mucopolysaccharidosis
ERT: Enzyme replacement treatment
DBS: Dried blood spot
HGMD: Human Genome Mutation Database
ACMG: American College of Medical Genetics and Genomics
GAG: Glycosaminoglycan
USG: Ultrasonography
