# Supplementary material for: α-mannosidosis diagnosis in Brazilian patients with MPS-like symptoms
Source: Orphanet J Rare Dis. 2024 Nov 26;19:439. doi: 10.1186/s13023-024-03419-z (PMC11600758; doi:10.1186/s13023-024-03419-z)
Supplement: Supplementary file 1 — Supplementary Material 1 [file 13023_2024_3419_MOESM1_ESM.docx]

SUPPLEMENTARY MATERIAL_TABLES S1, S2 and S3.

Table S1. Clinical description and manifestations from the 53 patients screened for α-mannosidase activity in the DBS and submitted to the MAN2B1 gene sequencing.

| Family ID | Patient code | Origin (Brazilian state) | Gender | Age | Clinical manifestations |
| --- | --- | --- | --- | --- | --- |
| A | P1 | PI | M | 22 yo | Developmental delay, hepatosplenomegaly, short stature and bone changes. Consanguineous parents. |
| B | P2 | PB | M | 6 yo. | Characteristic signs of the suspected syndrome. |
| C | P3 | SP | M | 34 yo | Neurodevelopmental regression and hepatosplenomegaly. |
| C | P4 | SP | F | 29 yo | Learning disability, ataxic-hemiparetic, coordination impairment and heteroagressivity and neurologic regression. |
| D | P5 | SP | M | 5 yo | Skeletal changes and indented vertebrae. |
| F | P6 | AL | M | 11 yo | Hepatosplenomegaly, frequent infections of the respiratory tract, joint restriction, cardiovascular palpitations, cognitive impairment and aggressiveness. Consanguineous parents. |
| G | P7 | AL | M | 14 yo | Hepatosplenomegaly, frequent infections of the respiratory tract, joint restriction, cardiovascular palpitations, cognitive impairment and aggressiveness. Consanguineous parents. |
| H | P8 | AL | M | 10 yo | Characteristic signs of the suspected syndrome. |
| I | P9 | MG | M | 4 yo | Recurrent otitis media, nasal obstruction, facial dysmorphia, enlarged protruding tongue and lips. |
| J | P10 | PB | M | 12 yo | Characteristic signs of the suspected syndrome. |
| K | P11 | PB | M | 7 yo | Characteristic signs of the suspected syndrome. |
| L | P12 | PB | M | 16 yo | Characteristic signs of the suspected syndrome. |
| M | P13 | PB | M | 20 yo | Characteristic signs of the suspected syndrome. |
| N | P14 | MG | M | 3 yo | Umbilical hernia, claw hands, joint stiffness, "gibbus" deformity, growth retardation, cognitive impairment, heart murmur, hepatomegaly, and delayed development of teeth. |
| O | P15 | SE | M | 2 yo | Coarse facial features, recurrent respiratory tract infections, dystosis and neuropsychomotor developmental delay. |
| P | P16 | SC | M | 18 yo | Short stature, corneal clouding, glaucoma. |
| Q | P17 | SP | M | 1 yo | Skeletal changes, ocular impairment, renal failure, cardiovascular abnormalities, neurological manifestations, extremities and bone pain. |
| R | P18 | SP | M | 6 yo | Characteristic signs of the suspected syndrome. |
| S | P19 | SP | M | 65 yo | Skeletal defects, ocular impairment and bone pain. |
| T | P20 | SP | F | 12 yo | Characteristic signs of the suspected syndrome. |
| U | P21 | SP | M | 10 yo | Characteristic signs of the suspected syndrome. |
| V | P22 | SP | F | < 1 yo | Short neck and joint restriction. |
| W | P23 | MG | M | < 1 yo | Hyperthyroidism, hypotonia, facial dysmorphism with frontal hypertrichosis, enlarged nasal base, warhead palate, fugiform fingers and swallowing disorders. |
| X | P24 | SP | M | < 1 yo | Immune deficiency, hyperactivity and tonsillectomy. |
| Y | P25 | SP | M | 7 yo | Epilepsy, hyperactivity and brain MRI altered. |
| Z | P26 | SP | M | 8 yo | Epilepsy, sleep disorder, mouth breather, cognitive deficit and tonsillectomy. |
| A1 | P27 | SP | M | 19 yo | Carpal tunnel syndrome, short stature, epilepsy and tonsillitis. |
| A2 | P28 | SP | M | 11 yo | Adenoidectomy and tonsillectomy. |
| A3 | P29 | SP | M | 12 yo | Cognitive impairment, mouth breather and tonsillitis. |
| A4 | P30 | SP | M | 12 yo | Cognitive impairment, adenoidectomy, tonsillectomy and gastroesophageal reflux disease. |
| A5 | P31 | SP | M | 9 yo | Mouth breather, adenoidectomy and tonsillitis. |
| A6 | P32 | SP | M | 10 yo | Apnea, adenoidectomy and tonsillectomy. |
| A7 | P33 | SP | M | 7 yo | Hypotonia, seizure, adenoidectomy, tonsillectomy and  neuropsychomotor developmental delay. |
| A8 | P34 | SP | M | 12 yo | Mouth breathing, otitis and cognitive deficit. |
| A9 | P35 | SP | M | 10 yo | Malformation, adenoids and tonsils. |
| A10 | P36 | SP | M | 8 yo | Apnea, adenoid and tonsil surgery, hyperactivity and short stature. |
| A11 | P37 | SP | F | 8 yo | Cognitive impairment, short stature and bone dysplasia. |
| A12 | P38 | SP | M | 6 yo | Short stature, dysmorphism, otitis and bone dysplasia. |
| A13 | P39 | SP | M | 6 yo | Autism, cognitive impairment and malformation. |
| A14 | P40 | PB | M | < 1 yo | Characteristic signs of the suspected syndrome. |
| A15 | P41 | MG | M | 2 yo | Characteristic signs of the suspected syndrome. |
| A16 | P42 | SP | F | 11 yo | Craniostenosis. |
| A17 | P43 | SP | F | 25 yo | Short stature, platyspondilia, bone dysplasia. consanguineous parents. |
| A18 | P44 | SP | M | 50 yo | Short stature, platyspondilia, bone dysplasia and malformation. |
| A19 | P45 | SP | M | 9 yo | Heart disease, short stature and bone changes. consanguineous parents. |
| A20 | P46 | SP | M | 6 yo | Hepatomegaly, short stature and mild dysformia. consanguineous parents. |
| A21 | P47 | SP | M | 1 yo | Hypotonia, neurological impairment and skeletal changes. |
| A22 | P48 | SP | F | 2 yo | Hypotonia and neurological impairment. |
| A23 | P49 | SP | M | 1 yo | Neurological impairment. |
| A24 | P50 | SP | M | 1 yo | Characteristic signs of the suspected syndrome. |
| A25 | P51 | SP | M | 2 yo | Hepatosplenomegaly, neurological impairment and skeletal changes. |
| A26 | P52 | MG | F | 39 yo | Characteristic signs of the suspected syndrome. |
| A27 | P53 | SP | F | 14 yo | Characteristic signs of the suspected syndrome. |

Legends:

F: female. M: male. N/I: not informed.

Brazilian States: PB: Paraíba, SP: São Paulo, AL: Alagoas, MG: Minas Gerais. SC: Santa Catarina, SE: Sergipe, PI: Piauí.

Table S2. PCR and sequencing primers list.

| Fragment Size  (pb) | Forward Primer | | Reverse Primer | *MAN2B1* gene region |
| --- | --- | --- | --- | --- |
| 561 | (1F) AGAGGCATTCCGTCTTCATAGC | | (1R) TCGCAATGACACAAAGCACG | Exon 1 |
| 690 | (2F) CTCCTCTGCACATAGGCGAG | | (2R) GCGACACGCATGTTATACAGC | Exons 2 and 3 |
| 576 | (3F) GTGCAAGCTGTATAACATGCG | | (3R) CCTGGTCCTTGTGAGATTGC | Exon 4 |
| 676 | (4F) AAGCAGAGGGGAGTTTGGTG | | (4R) AGAACGTGTTAGGGGATGCC | Exons 5 and 6 |
| 450 | (5F) GTGGGTCCAAGAGAACTGC | | (5R) GAGCTGCTAAGTGTGTGGTC | Exon 7 |
| 848 | (6F) GGTAACAATCTGACAGTCCTGC | | (6R) TAGGGGTGGTTTCCAACTTC | Exons 8, 9 and 10 |
| - | (6.1F) GTAGGTCAGTGAAACATGACGAC* | | - | Exons 9 and 10 |
| 592 | (7F) GTCTCGATTTGTCTCCCAGGC | | (7R) GCCTCACACCTGTCTCTGTC | Exon 11 |
| 901 | (8F) GGACAGAGACAGGTGTGAGG | | (8R) GGTATTCATGGAAGCAGGCG | Exons 12 and 13 |
| - | (8.1F) ACAGCTAAACATCAGCATCTGC* | | - | Exon 13 |
| 733 | (9F) CCTGCTGGATTATAGGCATGAGC | | (9R) TGCTCAAGGACACACAATGAGG | Exon 14 |
| 713 | (10F) ACTTGGATAGCTGAGGCACG | | (10R) GGAGACTCTCATACGACCAGC | Exon 15 and 16 |
| - | (10.1F) CACAAACCCATCTGTGGACC* | - | | Exons 15 and 16 |
| 528 | (11F) CGGTGACTGAAGGTTGACC | | (11R) CTCCCACACTCATGTAATCAGC | Exons 17 and 18 |
| 800 | (12F) AGGACGAAGGGAGAGTGAAGG | | (12R) AACGGAGTCCTCAGCTTAGTGG | Exons 19 and 20 |
| - | (12.1F) GCCAGATCCCAAGCCTGATC* | | - | Exons 19 and 20 |
| 475 | (13F) CTTCATCCTCATCCGTTCCTC | | (13R) GGTTTGGGGCTAATTATGGC | Exon 21 |
| 545 | (14F) CGCCGTAACTCCCAAAGTCTC | | (14R) GATGGTGAAGGTGGAGAACAGG | Exon 22 |
| 900 | (15F) GAGAAGGAGATCGGAGAGAGGC | | (15R) AGCGTTTTAATGGCAGCAGC | Exons 23 and 24 |
| - | (15.1F) GGTCAGATTGAGGGTACGAGG* | | (15.1R) CTAACCTGGGTCTGGACTCTGC* | Exons 23 and 24 |

*primers used only for sequencing.

Table S3. PCR conditions for amplification of *MAN2B1* gene fragments.

| Temperature | Time | Cycles |
| --- | --- | --- |
| 95ºC | 5 min | 1x |
| 95ºC | 45 s | 35x |
| 61ºC | 30 s | 35x |
| 72ºC | 55 s | 35x |
| 72ºC | 10 min | 1x |
| 16ºC | ∞ |  |
